# The New Munich Elbow Soft Tissue (MEST) classification is a reliable MRI‐based injury severity grading system for acute simple elbow dislocations

**DOI:** 10.1002/ksa.70505

**Published:** 2026-07-13

**Authors:** Anja M. Wackerle, Romed P. Vieider, Pavel Kadantsev, Alexander Marka, Yannik Leonhardt, Marie Kluge, Lucca Lacheta, Sebastian Siebenlist

**Affiliations:** ^1^ Department of Sports Orthopaedics Technical University of Munich Munich Germany; ^2^ Department of Radiology Technical University of Munich Munich Germany

**Keywords:** classification, elbow, musculotendinous injury, reliability, simple elbow dislocation

## Abstract

**Purpose:**

Simple elbow dislocation (SED) is characterized by injuries to soft tissue structures. The aim of the novel Munich Elbow Soft Tissue (MEST) classification is to provide a systematic approach to grade the severity of musculoligamentous lesions after acute SED. The purpose of this study is to determine the reliability of the classification.

**Methods:**

All elbow dislocations between 1 January 2012 and 31 December 2024 that presented to a high‐frequency, multilevel trauma centre were screened retrospectively for inclusion criteria. Patients with a history of elbow fractures, chronic instability, previous surgery, previous musculoligamentous injuries, time from injury to magnetic resonance imaging (MRI) > 30 days and a lack of or low‐quality MRI imaging were excluded. In 30 randomly selected patients, injuries were classified according to the MEST system into Grade 1 (ligamentous) and Grade 2 (musculoligamentous), with an extended subclassification comprising eight subtypes based on detailed structure‐specific MRI assessment. Inter‐ and intrarater reliability were evaluated using Fleiss' and Cohen's Kappa (*κ)*.

**Results:**

Of the randomly selected 30 patients (age 38.1 ± 13.4 years, male 46.7%), the mean time from injury to MRI was 4.2 ± 3.9 days. Interrater reliability across all raters demonstrated substantial agreement for the MEST grade (Fleiss' *κ* 0.690) and for the MEST subclassification (Fleiss' *κ* 0.639). Exact agreement was found in 70.0% for the MEST grade and in 43.3% for the MEST subclassification. Agreement between radiologists yielded higher agreement for the MEST subclassification than between the orthopaedics, and vice versa for the MEST grade. The less experienced raters showed higher agreement than the experienced raters. The classification also showed substantial to excellent intrarater reliability, with mean *κ* values of 0.900 for the MEST grade and 0.800 for the MEST subclassification.

**Conclusion:**

The MEST classification represents a comprehensive and reliable classification for injury patterns after acute SED, offering additional guidance in clinical decision‐making.

**Level of Evidence:**

Level IV.

**Abbreviations**
BMI: body mass index
LUCL: lateral ulnar collateral ligament
MCL: medial collateral ligament
MEST: Munich Elbow Soft Tissue
MRI: magnetic resonance imaging
SED: simple elbow dislocation

## INTRODUCTION

Simple elbow dislocations (SED) are characterized as elbow dislocations without concomitant fractures and represent one of the most common traumatic injuries of the elbow joint [6, 9]. Depending on the injury mechanism, different distribution patterns of musculoligamentous injuries after acute SED have been described [1, 4, 16].

In addition to clinical examination, magnetic resonance imaging (MRI) is the preferred imaging modality for assessing the extent of soft tissue injuries following acute SED, although its diagnostic accuracy and reliability remain subjects of ongoing debate [4, 5, 18]. Despite the clear clinical importance of musculoligamentous injuries after SED, there is a scarcity of standardized MRI‐based classification systems. As a result, clinicians relied on subjective interpretation when assessing diagnostic findings and making treatment decisions.

The previously published Munich Elbow Soft Tissue (MEST) Classification was developed to address this gap in clinical practice. The MEST classification is a solely MRI‐based classification system for musculoligamentous injuries after acute SEDs [10]. It distinguishes between two grades of severity of musculoligamentous injuries (isolated ligamentous injuries vs. musculoligamentous injuries) with subtypes based on the location of the main pathology (medial vs. lateral vs. bilateral). Given the numerous and varied combinations of musculoligamentous injuries following SED, it is crucial to assess the reliability of a proposed classification system [8, 13].

Therefore, the purpose of this study was to evaluate the comprehensiveness, the interrater and intrarater reliability of the MEST classification. It was hypothesized that the MEST classification provides a reliable categorization of the different musculoligamentous injury patterns after acute SEDs.

## MATERIALS AND METHODS

### Imaging selection

All elbow dislocations that presented to the senior authors' high‐frequency, multilevel trauma centre between 1 January 2012 and 31 December 2024 were screened for inclusion retrospectively. Patients with acute or a history of fractures, chronic instability, previous elbow surgery or previous musculoligamentous injuries of the elbow joint, and patients without available MRI imaging or MRIs with a time of injury to MRI > 30 days were excluded. In total, 94 patients met the inclusion criteria and a randomly selected subset of 30 patients was used to assess inter‐ and intrarater reliability analysis.

### MRI evaluation

#### Raters

The included MRIs were examined by a total of four blinded raters: two radiologists with a focus on musculoskeletal radiology and two orthopaedic surgeons with a focus on elbow surgery. All 30 MRI examinations were performed using 3.0 Tesla scanners. Imaging was not limited to a single scanner or institution, reflecting the heterogeneity of real‐world clinical practice. The raters' experience and the average number of weekly‐assessed MRI examinations of them are shown in Table 1. The raters were blinded to the diagnosis and treatment of the patients.

**Table 1:** Summary of specialty and experience of all four raters.

Rater	O1	O2	R1	R2
Specialty	Orthopaedics	Orthopaedics	Radiology	Radiology
Clinical position	Specialist	Resident	Specialist	Resident
Experience evaluating musculoskeletal MRI (years)	10	4	6	3
Experience evaluating elbow MRI (years)	10	4	5	3
Number of evaluated musculoskeletal MRIs per week over the last year	60	45	70	45
Number of evaluated elbow MRIs per week over the last year	20	10	5	3

Abbreviation: MRI, magnetic resonance imaging.

### Rating protocol

Injuries to the medial and lateral musculoligamentous structures were evaluated on MRI. All raters independently assessed the integrity of the medial collateral ligament (MCL), the lateral ulnar collateral ligament (LUCL), the common extensor origin and the common flexor origin. Structures were rated as either intact (<50% of tissue with lesion) or not intact (defined as ≥50% of tissue with lesion). All raters analysed the MRIs at a minimum of 4 weeks after the first rating for a second time.

### MEST classification

The MEST classification is an MRI‐based system for acute SEDs, distinguishing two grades of injury severity of musculoligamentous injuries [10]. Grade 1 comprises medial (1m), lateral (1l) or bilateral (1lm) ligamentous injuries. Grade 2 includes combined musculotendinous injuries, including the flexor tendons on the medial side (2M) or extensor tendons on the lateral (2L) side or bilateral (2LM) musculotendinous injuries [10].

In the present study, a detailed, structure‐specific MRI analysis of the MCL, LUCL, flexor and extensor tendons was performed. This revealed recurring combined injury patterns within Grade 2 that were not fully captured by the original subclassification (Figure 1). To better represent these combinations, the classification was extended by introducing two additional subclasses: combined injuries of MCL, LUCL and flexors (2Ml) and of MCL, LUCL and extensors (2Lm). As a result, Grade 2 injuries are subdivided into five categories (2M, 2L, 2ML, 2Ml and 2Lm), allowing for a more precise characterization of injury patterns.

**Figure 1:**
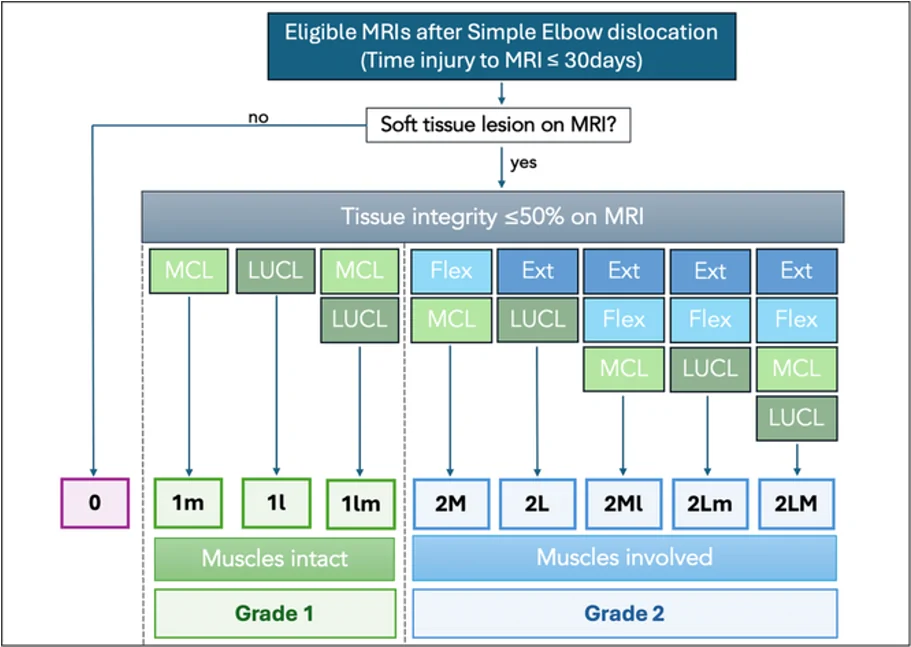
Overview of the modified MEST classification system. Simple elbow dislocations (SEDs) with soft tissue lesions on MRI are classified into MEST Grade 1 (green) and 2 (blue), and further subclassified depending on the injury involvement of the medial collateral ligament (MCL), the lateral ulnar collateral ligament (LUCL), the flexor muscles (Flex) and the extensor muscles (Ext) into a total of eight injury subgroups. MEST, Munich Elbow Soft Tissue classification; MRI, magnetic resonance imaging.

### Statistical analysis

To characterize the study population, descriptive statistics, including mean, range, absolute frequencies and relative frequencies, are reported. An analysis was conducted on the interobserver agreement among all raters and several subgroups (radiologists, orthopaedic surgeons, experienced, non‐experienced). The raters with experience in evaluating musculoskeletal MRIs for 5 years or more were defined as experienced (Radiologist 1 and Orthopaedic surgeon 1), while those with less than 5 years of experience were labelled as non‐experienced examiners (Radiologist 2 and Orthopaedic surgeon 2).

Interrater reliability was evaluated using Fleiss' *κ* across all four raters for each anatomical parameter (MCL, LUCL, extensors, flexors), the overall MEST grade (1 vs. 2) and the specific MEST subgroups. In addition, Cohen's *κ* was calculated between raters of the same specialty (R1 vs. R2; O1 vs. O2) and between raters of the same experience level (R1 vs. O1; R2 vs. O2), all at Time point 1. Intrarater reliability was assessed with Cohen's *κ* by comparing ratings from Time point 1 and Time point 2 for each rater, applied to all anatomical parameters, the overall MEST grade and the specific MEST subgroups.

## RESULTS

Of the 30 patients included in the analysis, the mean age at time of SED was 38.1 ± 13.4 years ([body mass index, BMI = 24.8 ± 3.2, female: *n* = 16 [53.3%]) (Table 2). The left elbow was affected more frequently (73.3%) than the right (26.7%), which was statistically significant (*p* < 0.05). Mean time from injury to MRI was 4.2 ± 3.9 days (range, 0–17 days).

**Table 2:** Summary of demographics of all included patients.

Demographics	
Age when SED was diagnosed, years	38.1 ± 13.4
Female sex, *n* (%)	*n* = 16 (53.3%)
BMI	24.8 ± 3.2
Affected elbow laterality left, *n* (%)	22 (73.3%)
Time from injury to MRI, days	4.2 ± 3.9

Abbreviations: BMI, body mass index; MRI, magnetic resonance imaging; SED, simple elbow dislocation.

Interrater reliability across all four raters demonstrated substantial agreement for the evaluation of individual anatomical structures, with Fleiss' *κ* ranging from 0.661 for the extensors to 0.782 for the LUCL. For the MEST classification, Fleiss' *κ* was 0.690 for the overall grade and 0.639 for the subgroup classification. Exact agreement of all raters was found in 70.0% for the overall MEST grade and in 43.3% for the MEST subclassification.

When stratified by specialty, agreement between the two radiologists yielded Cohen's *κ* values ranging from 0.634 (LUCL) to 0.870 (MCL) for the anatomical structures, 0.777 for the overall MEST grade and 0.673 for the MEST subgroups. Agreement between the two orthopaedic surgeons ranged from 0.634 (LUCL) to 0.783 (MCL) for anatomical structures, with *κ* values of 0.800 for the overall MEST grade and 0.633 for the subgroup classification (Table 2).

Comparison of raters with similar levels of experience showed consistent results. Between the senior raters (R1 and O1), *κ* values ranged from 0.661 (flexors) to 1.0 (LUCL) for the anatomical structures, 0.638 for the overall MEST grade and 0.632 for the MEST subgroups. Between the junior raters (R2 and O2), agreement ranged from 0.577 (extensors) to 0.857 (flexors), with *κ* values of 0.733 for the overall MEST grade and 0.635 for the subgroups (Table 3).

**Table 3:** Results of the interrater agreement (*κF* = Fleiss *κ*, *κC* = Cohen's *κ*).

Interrater agreement	All raters (*n* = 4) *κ_F_*	Orthopaedics (*n* = 2) *κ_C_*	Radiologists (*n* = 2) *κ_C_*	Experienced raters (*n* = 2) *κ_C_*	Less experienced raters (*n* = 2) *κ_C_*
Anatomical structures					
MCL	0.730	0.783	0.870	0.714	0.634
LUCL	0.782	0.634	0.634	1	0.634
Flexors	0.755	0.780	0.730	0.661	0.857
Extensors	0.661	0.658	0.670	0.735	0.577
MEST grade	0.690	0.800	0.777	0.638	0.733
MEST subgroup	0.639	0.633	0.673	0.632	0.635

Abbreviations: LUCL, lateral ulnar collateral ligament; MCL, medial collateral ligament; MEST, Munich Elbow Soft Tissue classification.

Intrarater reliability was consistently higher than interrater agreement. Across all raters, mean intrarater *κ* values ranged from 0.820 (extensors) to 0.970 (MCL) for the anatomical structures (Table 4). The MEST classification also showed excellent intrarater reliability, with mean *κ* values of 0.900 for the overall grade and 0.800 for the subgroup classification.

**Table 4:** Results of the mean intrarater agreement (*κC* = Cohen's *κ*).

	MCL (*n* = 4) *κ*_*C*_	LUCL (*n* = 4) *κ*_*C*_	Flexors (*n* = 4) *κ*_*C*_	Extensors (*n* = 4) *κ*_*C*_	MEST Grade (*n* = 4) *κ*_*C*_	MEST subgroup (*n* = 4) *κ*_*C*_
Intrarater agreement	0.970	0.890	0.840	0.820	0.900	0.800

Abbreviations: LUCL, lateral ulnar collateral ligament; MCL, medial collateral ligament; MEST, Munich Elbow Soft Tissue classification.

## DISCUSSION

The most important finding of the current study was that the interrater and intrarater reliability of the MEST classification used in the diagnosis and classification of musculoligamentous injuries after acute SED was overall substantial. Interrater reliability between four raters showed substantial agreement, and intrarater reliability showed strong agreement between two time points.

The injury mechanism in SED is often described as a hyperextension injury mechanism [11]. Osborne and Cotterill proposed that the primary injury causing elbow instability and dislocation was the failure of the posterolateral ligamentous and capsular structures [16]. O'Driscoll et al. described a circular progression of ligamentous injury during elbow dislocation, known as the ‘Horii Circle’, which follows a chronological sequence of soft tissue disruption from lateral to medial [14, 15]. However, other authors have suggested the failure sequence starts on the medial side with subsequent variable energy dissipation laterally [19]. Despite the various injury mechanisms described, the associated injury patterns vary significantly and may complicate comparison between patients due to the heterogeneous combinations of musculoligamentous injury patterns after SED [12, 17, 20]. The complexity of soft tissue lesions, which include ligamentous disruptions and musculotendinous lesions, can lead to persistent instability, stiffness and chronic pain [2, 9].

A comprehensive classification system allows for categorization at the time of injury and may help in predicting outcomes or treatment decisions. A key challenge in developing a new classification system is ensuring its consistent application by different raters. Variability in MRI interpretation is a known issue in musculoskeletal imaging, influenced by factors such as differences in experience, training background and imaging protocol variations. Inferior levels of interrater agreement on structure integrity have been reported specifically for elbow MRI [18]. The results of the current study provide substantial agreement of the MEST classification between a total of four raters of two specialties. Previously published MRI classifications, considering for example the shoulder joint, reported similar agreement and are widely used in clinical practice [13].

MRI has previously been reported to be a valuable diagnostic tool for detecting full‐thickness lesions of the collateral ligaments in chronic elbow instability [21]. The subgroup analysis of the current study showed that more experienced raters demonstrated the highest agreement strength for the LUCL, whereas less experienced raters reached the highest agreement for flexor integrity. Across all raters, the strongest agreement was achieved for the LUCL, followed by the flexor muscles, MCL and finally the extensor muscles, which exhibited the lowest agreement. These findings are consistent with prior literature, which has shown that interrater reliabilities for joint structures of the elbow on MRI are generally fair to moderate for ligamentous and osteochondral injuries, with *κ* values most commonly in the range of 0.25–0.63, depending on the structure and severity of the lesion. For example, the evaluation of the LUCL and MCL yields *κ* values of 0.44 (LUCL) and 0.28 (MCL) among raters, depending on examiner experience and specialty [18]. For LUCL injuries, interrater reliability has been reported to be higher for full‐thickness tears in the midsubstance and distal locations (*κ* ≈ 0.55–0.63), but lower for partial‐thickness tears and for tissue characteristics such as thickening or calcification (*κ* often <0.2) [3].

In the present study, overall rater agreement reached a substantial level and was comparable to the agreement values previously reported for isolated structures of the elbow. This finding underscores the robustness of our results, particularly given the heterogeneous backgrounds and varying levels of experience among raters. Importantly, while prior studies have specifically focused on ligamentous structures, the results of the current study demonstrate that a comprehensive assessment of soft‐tissue injury patterns can achieve substantial reproducibility The lack of a standardized classification system for soft‐tissue lesions following SEDs has been challenging the comparison of injury patterns after acute SED. The recently proposed MEST classification provides a structured framework for the evaluation of soft‐tissue injuries in this context [10]. As high rates of concordance have been shown between MRI after SEDs and surgical findings (87.5% and 90.9% for MCL and LUCL, respectively) [5], an MRI‐based grading system with treatment recommendations can be highly relevant for clinical decision‐making. The findings of this study indicate that the MEST classification can be applied effectively across raters of different specialties and experience levels, yielding substantial interrater agreement and strong intrarater agreement. This supports its potential as a standardized tool for both clinical decision‐making and research applications in the management of acute SEDs.

This study has several limitations. No distinction was made between complete and partial ligament tears. Interpretation of MRI may be inherently difficult, as patients with acute elbow dislocation are often unable to fully extend the joint, preventing adequate tensioning of the collateral ligaments, potentially complicating the interpretation of their integrity [7, 19]. To reduce bias, only high‐quality MRI scans were included, which may limit the generalizability of our findings to routine clinical practice, where imaging quality is often lower. Imaging was not limited to a single scanner or institution; however, this reflects the heterogeneity of real‐world clinical practice.

## CONCLUSION

The MRI‐based classification system to describe musculoligamentous injuries after SED (MEST classification) is a reliable grading system to systematically define injury severity. Interrater reliability was substantial between raters of different specialties and levels of experience.

## AUTHOR CONTRIBUTIONS

All authors contributed to the study conception and design. The first draft of the manuscript was written by Anja M. Wackerle and Romed P. Vieider. All authors commented on previous versions of the manuscript. All authors read and approved the final manuscript. Project administration was done by Anja M. Wackerle, Romed P. Vieider, Marie Kluge and Lucca Lacheta.

## CONFLICT OF INTEREST STATEMENT

Sebastian Siebenlist is in a relationship with Arthrex GmbH, KLS Martin Group, Medartis AG and medi GmbH that includes consulting or advisory. The remaining authors declare no conflicts of interest.

## ETHICS STATEMENT

Ethical approval for this study was obtained from the Ethics Committee of the Technical University of Munich (2023‐448‐S‐SB). The study was approved by the local Institutional Review Board/Ethics Committee (approval no. (2023‐448‐S‐SB)). Owing to the retrospective design, patients provided general consent for the use of their anonymized clinical data for research purposes in accordance with institutional policy and the Declaration of Helsinki.

## Data Availability

The authors have nothing to report.
